# Optimizing Metapopulation Sustainability through a Checkerboard Strategy

**DOI:** 10.1371/journal.pcbi.1000643

**Published:** 2010-01-22

**Authors:** Yossi Ben Zion, Gur Yaari, Nadav M. Shnerb

**Affiliations:** 1 Department of Physics, Bar-Ilan University, Ramat-Gan, Israel; 2 Department of Ecology and Evolutionary Biology, Yale University, New Haven, Connecticut, United States of America; Wilfrid Laurier University, Canada

## Abstract

The persistence of a spatially structured population is determined by the rate of dispersal among habitat patches. If the local dynamic at the subpopulation level is extinction-prone, the system viability is maximal at intermediate connectivity where recolonization is allowed, but full synchronization that enables correlated extinction is forbidden. Here we developed and used an algorithm for agent-based simulations in order to study the persistence of a stochastic metapopulation. The effect of noise is shown to be dramatic, and the dynamics of the spatial population differs substantially from the predictions of deterministic models. This has been validated for the stochastic versions of the logistic map, the Ricker map and the Nicholson-Bailey host-parasitoid system. To analyze the possibility of extinction, previous studies were focused on the attractiveness (Lyapunov exponent) of stable solutions and the structure of their basin of attraction (dependence on initial population size). Our results suggest that these features are of secondary importance in the presence of stochasticity. Instead, optimal sustainability is achieved when decoherence is maximal. Individual-based simulations of metapopulations of different sizes, dimensions and noise types, show that the system's lifetime peaks when it displays checkerboard spatial patterns. This conclusion is supported by the results of a recently published *Drosophila* experiment. The checkerboard strategy provides a technique for the manipulation of migration rates (e.g., by constructing corridors) in order to affect the persistence of a metapopulation. It may be used in order to minimize the risk of extinction of an endangered species, or to maximize the efficiency of an eradication campaign.

## Author Summary

No one can produce all his needs by himself. Personal autarky poses a serious danger of collapse in cases of illness, drought, etc. Trade reduces the impact of local catastrophes, thus increasing economic stability. However, the recent series of econo-crises revealed that globalization induces coherence among markets and jeopardizes their sustainability against global failures. Economists try to identify the optimal tariff that balances between the dangers of autarky and the risk of correlated failure. The same problem appears in ecosystems with a population divided among local habitat patches. “Optimal tariff” is translated to optimal migration rate: how should one manipulate connectivity among patches in order to achieve maximum sustainability? Recolonization of habitats that undergo extinction is essential for survival, yet a too strong dispersal leads to coherence and correlated extinction. Here we use individual-based models in order to find the optimal migration rate. We show that this optimum appears when the the system takes a spatial “checkerboard” pattern that maximizes the decoherence. The insights gleaned allow for improved policies for conservation of endangered species (optimizing the effect of corridors, predicting the impact of habitat fragmentation) and, on the other hand, eradication campaigns (like vaccination or pest control).

## Introduction

In recent years, many studies in the field of biodiversity maintenance were focused on spatially structured populations [1]–[14]. Of particular importance are Levins type metapopulations [1],[2],[15], where distinct subpopulations occupy spatially segregated patches of habitats connected by migration. The principle aim of our research is to understand the effect of spatial structure on the persistence of the population; this will allow one to predict the impact of habitat fragmentation, to suggest systematic reserve design strategies [16], and to forecast the effect of conservation corridors [3].

The population of an isolated patch is usually unstable, as demographic and environmental fluctuations may drive the colony to extinction. Migration among subpopulations allows recolonization of vacant habitat patches (turnover events) and reduces the risk of correlated extinction [2]. If the dynamic of a large, well-mixed population is stable, spatial segregation is always harmful. To avoid global extinction, one should increase the migration among patches to allow for a maximal “rescue effect” [17],. Can one desire too much of a good thing? Greater mixing, or even patch merging, is the optimal conservation strategy; this is the fundamental assumption behind the reserve design guidelines of Diamond [18], for example.

**Figure 1:**
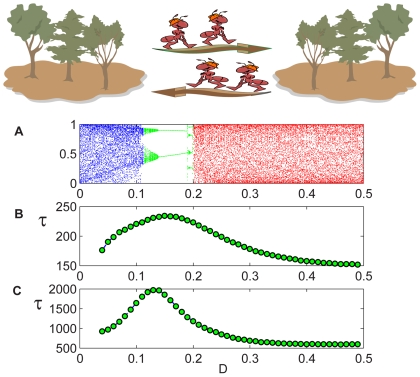
Coupled Logistic map, two-patch system. The dynamic is illustrated by a cartoon (upper panel). Intra-patch logistic growth is followed by a migration step; the graphs indicate the average lifetime 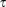 against the migration rate parameter 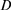. (A): The orbit (bifurcation) diagram for the deterministic system [Eq. (2)] with 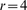. The total population follows a chaotic trajectory for either high migration (red region), where the patches synchronize, or for low dispersal rates (blue region), where each patch oscillates independently. In the middle region (green), a period-2 attractive “up-down” cycle appears, and the deterministic dynamic becomes stable. In this panel the y axis corresponds to all values 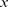 may take, which is, for the logistic map, the 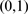 segment of the real line. (B) and (C): The average time-to-extinction of the individual-based dynamics [Eq. (1)] of the same system. The bell shape peaked at the values of 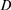 for which the up-down period-2 orbit appears in the deterministic map. Here 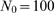 (B) and 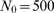 (C).

The situation becomes much more complicated if the local dynamics of a large, well-mixed population is also extinction-prone. In such a case, strong dispersal, which is equivalent to patch merging, increases spatial coherence and leads to global extinction. Many recent experiments on predator-prey [9]–[11],[6], host-parasite [5], and single species [12],[13] systems suggest that migration is a two-edged sword: it should not be too weak, so that it could allow for recolonization of empty patches by their neighbors, but if it becomes too large, the system synchronizes, the effect of local refuges is reduced, and all the patches undergo extinction together [3],[4]. The typical outcome is the “bell shape” demonstrated in panels b,c of Figure 1, where the average lifetime of a spatial stochastic system [the stochastic-logistic map, see Materials and Methods] is plotted against the (density-independent) migration rate. The left shoulder of the bell indicates an increase in the persistence with dispersal due to the rescue effect; along the right shoulder, migration becomes harmful as it leads to coherence and correlated extinction. Similar observations have been reported in several fields, ranging from evolutionary game theory [19] to the way globalization induces coherence among economic markets thus jeopardizing their stability [20].

There is a substantial literature on the two edges of the bell shape: the extinction transition that takes place as migration becomes too small [2], [21]–[24] and the synchronization transition when the mixing exceeds some threshold value [9],[3],[25],[26]. Here we intend to identify where the peak of the curve is, i.e., under what parameters the system achieves *maximum sustainability* such that the chance of extinction is minimal. For this purpose, we have developed a numerical technique that allows one to consider the effect of demographic stochasticity and the possibility of extinction for a spatially structured population. To demonstrate the scope of our results, we have considered first the most studied system in the field, namely, the logistic map, and then two other paradigmatic systems: the Ricker map and the well-known Nicholson-Bailey host-parasitoid dynamics.

Our main result is the identification of the conditions for maximum sustainability. Surprisingly, it turns out that the optimal point for the stochastic system has nothing to do with the stability properties of the deterministic (noise-free) dynamics. Instead, it always appears when the spatial system arranges itself in a *checkerboard pattern*. In the following section, we show that the maximal persistence time appears when the decoherence peaked, as this is the underlying mechanism beyond stability. The three different systems (logistic, Ricker, and Nicholson-Bailey) are analyzed in detail, and we demonstrate consistently that in each of them the maximum sustainability is associated with a checkerboard pattern.

Along this paper we deal solely with demographic stochasticity. However, it should be emphasized that our results hold in the presence of other types of noise, like the environmental stochasticity considered by [27],[28],[29] - see Text S1. The checkerboard strategy breaks down only when the population size is unrealistically high (in which case the system follows its deterministic dynamics) or extremely low (where the question of coherence among patches is irrelevant, see Materials and Methods).

## Results

### The Logistic Map

First let us present the numerical technique used in order to study the effect of demographic stochasticity on the sustainability of a spatially segregated population. We demonstrate this technique for the logistic system; the generalization of this method to any other dynamics is presented in the Materials and Methods section.

We consider a metapopulation with 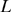 local habitat patches, where the carrying capacity of a patch is 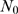. The dynamics is described by a discrete generation island model: local population of size 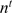 at time 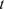 produces 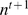 local individuals in the next generation. Any agent may then decide to emigrate from its local habitat with probability 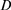; upon migration it chooses its destination with equal probability among 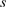 possible habitat patches.

Each of the 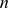 individuals in a local community produces 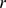 offspring, but the chance of an offspring to survive local competition is 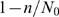, and thus the total population by the time of the next generation is *on average* 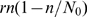. To consider demographic stochasticity we utilized the fact that 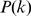, the probability that 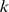 individuals (out of 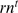) survive to the next generation is given by the binomial distribution,
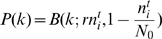
(1)where B(k;n,p) is the chance to get exactly 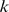 successes in 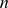 trials, if the chance of success in an individual trial is 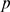. To avoid the possibility of an increase of the local community above 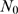 we impose 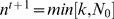.

Indeed, 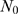 controls the strength of demographic stochasticity. If the population density 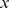 is defined as the rescaled number of individuals, 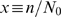, it is clear that the map 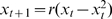 describes the dynamics of the average density for the stochastic process (1). Moreover, since the variance is proportional to the population size, the stochastic map converges to the deterministic one in the limit 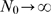, with fluctuations that scale like 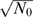 [30].

The deterministic limit of the stochastic-logistic system corresponds, thus, to the paradigmatic model of diffusively coupled logistic maps, considered already in the context of population dynamics [3]. In its spatially explicit form, this system obeys (here 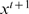 is the population density after the dispersal/migration step):
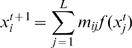
(2)where 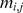 is the proportion of individuals from patch 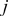 that disperse to patch 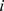, where 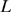 is the number of patches, 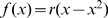, and where 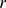 is the maximal intrinsic growth rate (maximum fecundity) of the population. In this work, we have considered only local dynamics, where 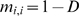 and 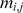 is zero unless the 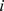 and the 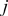 sites are nearest neighbors, in which case 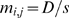, where 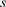 is the connectivity of the 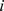
th site.

Note that this deterministic model *per se* may support chaotic dynamics where the population assumes an arbitrarily small value, but it never allows for extinction. To consider the possibility of extinction, one must adds demographic stochasticity to the model, i.e, use (1) instead of (2). For a small system, the average time to extinction 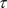 may be estimated by averaging over many runs with different initial conditions and different histories; this is the method used to obtain the graphs presented in the next figures. For large systems, or alternatively when the time to extinction is relatively large, we have used other estimation techniques (see the Materials and Methods section for the details of these techniques and a comparison between them.)

Figure 1 exemplifies the situation for the simplest case, namely, a two-patch system. In that figure, two models are presented: in the upper panel the deterministic dynamic of a coupled logistic map [Eq. (2)], and below it, two panels with different 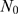 of its stochastic, agent based analog [described by Eq. (1)]. The orbit diagram of the deterministic map shows that, for some intermediate migration rate, the system supports an attractive, period-2 orbit [31],[32]. This orbit is characterized by an “up-down” dynamic: when one patch is “up” (admits a larger population) the second is “down” (in the low-density state) and vice-versa. Interestingly, the peak of the bell shape for the persistence time in the cases of the stochastic (agent-based) system happens to be in that same “up-down” region.

**Figure 2:**
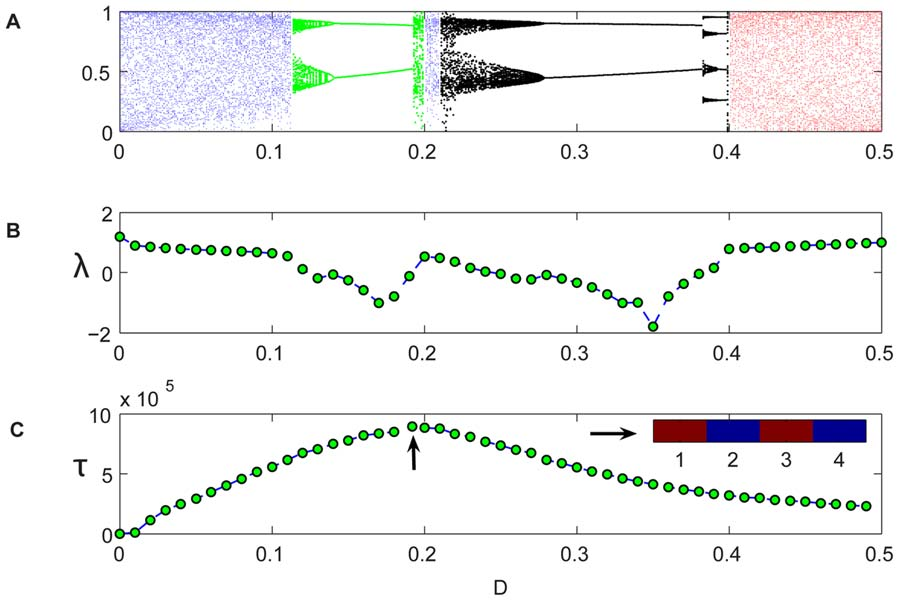
Coupled Logistic map, four-patch system with periodic boundary conditions. Here the deterministic (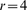) orbit diagram (A) shows two stable regions that correspond to alternating (up-down-up-down, green, and up-up-down-down, black) spatial configurations. Panel (B) shows the Lyapunov exponent 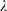 of the orbit: while the chaotic regions are characterized by a positive exponent indicating that the trajectories are unstable, for the periodic orbits, 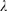 is negative. Yet, although the UUDD orbits are more attractive (more negative 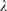), the lifetime of the stochastic system (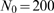) peaks in the UDUD region, as shown in panel (C). The vertical arrow shows where the time to extinction is maximal; the horizontal arrow points to a representative snapshot of the agent-based system in that optimal migration, while the colors represent density. Clearly, the maximal sustainability (smallest chance for extinction) occurs in the checkerboard (UDUD) phase.

Does this fact indicate that the peak should be *attributed* to the features of the spatial deterministic dynamics, namely, to the existence of an attractive manifold? Not really. Let us take a look at Figure 2. Here plots are given for a four-patch system with periodic boundary conditions (a square with no diagonal connections). There are two regions in the orbit diagram presented in the upper panel that correspond to period-2 attractive manifold. The first is the “up-down-up-down” (UDUD) region (two up-down patches attached to each other) and the second is an “up-up-down-down” (UUDD) configuration, where diffusion is strong enough to synchronize adjacent pairs. In the second panel, the Lyapunov exponent of the orbits is presented, and one finds that the UUDD is slightly more attractive than the UDUD region. However, as can be seen in the third panel, the peak of the persistence time is still found in the UDUD region, and the bell-shape is smooth, completely unaffected by the appearance of the UUDD periodic orbit. There is no direct correspondence between the appearance of attractive orbits of the deterministic map and the persistence of the stochastic system.

**Figure 3:**
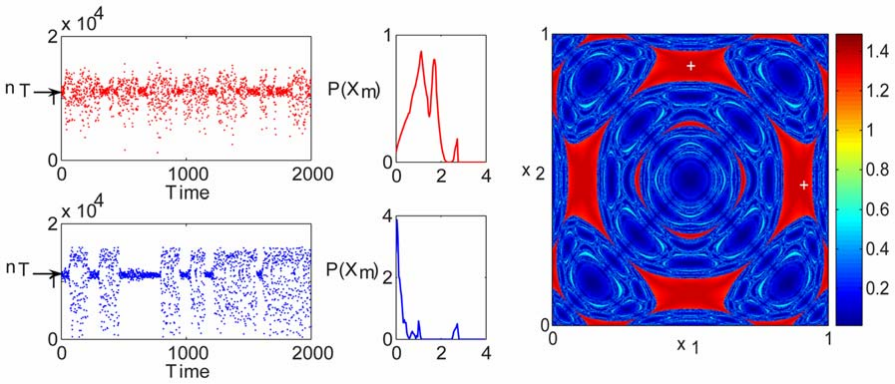
Effect of long excursions. The time evolution of total population (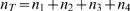) vs. time for the four-patch system of Figure 2 with individual based dynamics [Eq. (1)] (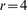 and 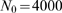) for 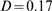 (UDUD region, left red) and 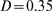 (UUDD region, left blue). Most of the time the system sticks to the population level that corresponds to the attractive orbit (indicated by an arrow), and these stability periods are indeed longer in the UUDD case, in agreement with its stronger attractiveness. However, from time to time the demographic noise drives the system out of the basin of attraction of the periodic orbit and then the system follows a long excursion until it again reaches the attractive orbits. The chance of extinction has nothing to do with the stability of the attractive orbits. Instead, it is determined by the point of lowest population during an excursion. In the right panel, this characteristic of the trajectory is demonstrated: for the deterministic two-patch system with 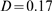, the period-2 orbit (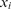 takes alternately the values 0.48 and 0.89, marked by crosses) is attractive, but with different initial conditions it undergoes completely different trajectories. The minimum value of the total population (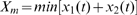) along the excursion from any starting point to the asymptotic states (crosses) is color-coded (see color bar) and yields a fractal map [32]. A histogram of 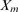 for the deterministic 4-patch configurations (middle panels) shows much larger support close to zero for the UUDD configurations, which explains why the checkerboard arrangement is more persistent.

Figure 3 explains why the analysis of stability using Lyapunov exponent is irrelevant for the prediction of the maximum sustainability point. The UDUD and the UUDD orbits are indeed attractive, but their basin of attraction is narrow, and small perturbations take the system to long excursions until it reaches the stable manifold again. [In the theory of nonlinear dynamics such systems are known as excitable [33], and the excursion defines a homoclinic trajectory]. What determines the chance of extinction is not the local stability properties of the orbits but the probability of extinction during the excursion. This chance is proportional to the minimum distance to default along the excursion path.

These individual particle simulations show that for realistic values of 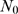, up to 5000 agents per site, demographic stochasticity is strong enough to kick the system occasionally from the attractive orbit, sending it to a long excursion in phase space. It turns out that since the underlying dynamics is chaotic, the kicked system visits any possible point in phase space with almost equal chance. There is no need to make a distinction between asymptotic states of different initial conditions: what matters is the minimal total population along the transient. Indeed one may simply average the distance to default over many initial conditions of the deterministic system to get roughly the same bell-shape obtained from the individual-based simulations (see Materials and Methods and Video S1).

The fractal basin boundary and the dependence of the asymptotic behavior on initial conditions have already been pointed out by Adler [34] for the Nicholson-Bailey map and by Hastings [32] for Ricker and logistic systems. Indeed, it is this feature of the deterministic model that makes stochasticity an important factor. In general, one may guess that there is no need to add stochasticity to the already random, erratic dynamics of a chaotic system, and on the other hand, that the effect of weak stochasticity on a system that admits an attractive orbit is small. Here we find that these two arguments fail when a stable orbit results from the interplay between chaotic subpopulations: stochasticity is still important and its little affect is amplified by the underlying chaotic motion that yield these long excursions.

### The Ricker Map and the Nicholson-Bailey Host-Parasitoid Model

#### The Ricker map

**Figure 4:**
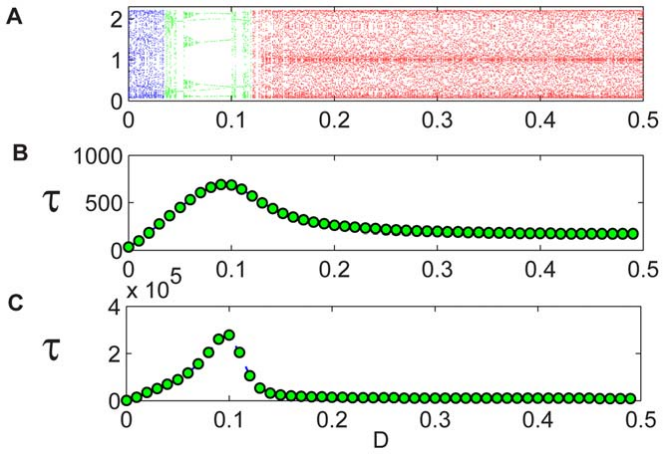
Coupled Ricker map, two-patch system. (A): The orbit diagram for the deterministic system [Eq. (7)] with 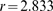. In the low-migration (blue) region the patches are independent; in the right (red) region they are synchronized. The intermediate migration (green) regime is characterized by up-down dynamics (not necessarily of period-2!). The average time-to-extinction of the individual-based dynamics [Eq. (10)] shows persistence peaks in this region for both 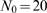 (B) and 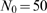 (C).

**Figure 5:**
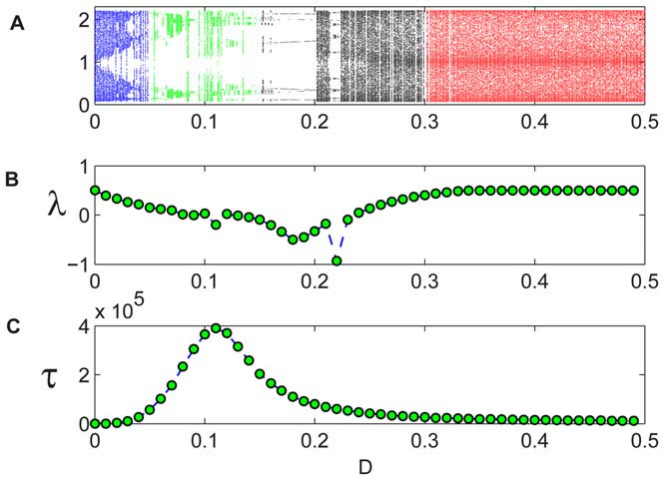
Coupled Ricker map, four-patch system. The orbit diagram (A) presented together with the Lyapunov exponent 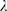 of the attractive orbit (B), shows that the up-up-down-down orbits (black region) are much more attractive than the up-down-up-down orbits (green). Yet the persistence peaks in the checkerboard region for 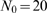 in (C).

Figure 4 shows the orbit diagram and the persistence curves for a two-patch Ricker map where the local dynamics obeys:
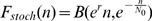
(3)as described in the Materials and Methods section. In Figure 5, the four-patch system stability is analyzed for the Ricker case, in parallel with the analysis presented in the logistic case.

#### The Nicholson-Bailey map

**Figure 6:**
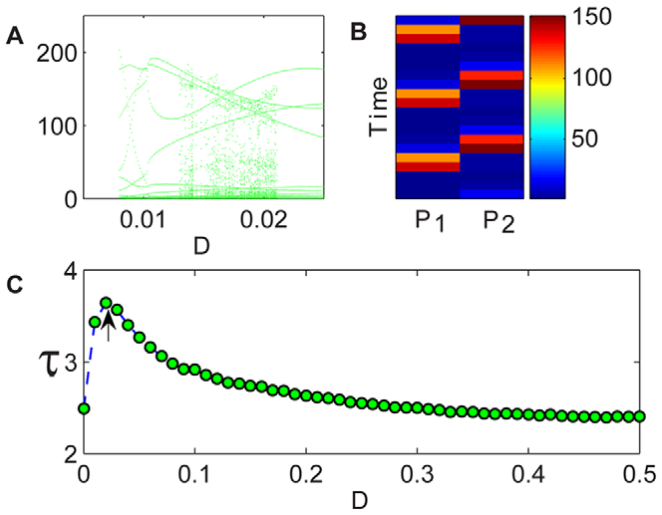
Nicholson-Bailey map, two-patch system. The orbit diagram (A) is presented only for the narrow range of parameters where the deterministic map [Eq. (11)] supports periodic orbits; in that region the persistence curve (C) of the individual-based dynamics [Eq. (12)] admits its maximum. In Panel (B), the population on patch 1 (P1) and 2 (P2) is given at different times for 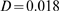, a value that corresponds to the optimal sustainability [indicated by an arrow in (C)]. Clearly the optimum corresponds to the up-down orbit.

**Figure 7:**
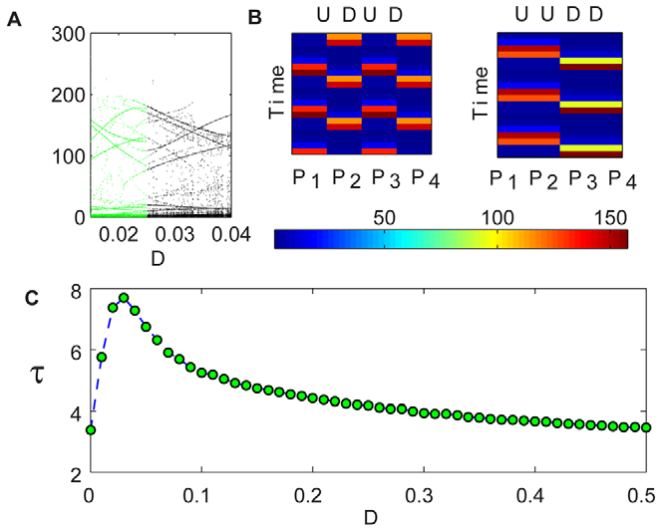
Nicholson-Bailey map, four-patch system. In the orbit diagram (A), the black region corresponds to the UUDD orbits, and the green to the UDUD. In the UDUD region the persistence curve (C) of the individual-based dynamics admits its maximum. In Panel (B), the population sizes on patch 1–4 is given at different times for 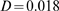 (UDUD), a value that corresponds to the optimal sustainability, and for 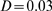 (UUDD). The color bar indicates the number of agents for panel (b).

Figures 6 and 7 show the stability properties of coupled Nicholson-Bailey maps:
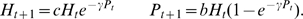
(4)


In the NB case we can show the orbit diagram for only a limited region of the phase space, where generic initial conditions converge to an attractive orbit, a phenomenon first pointed out by Adler [34]. Simulation results are presented for 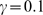, 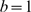 and 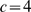.

In both the Ricker and the Nicholson-Baily maps, the system achieves maximum sustainability in the “UDUD” region, similarly to the case of the logistic map. Here too, the value of the Lyapunov exponent turns out to be irrelevant for determining the maximum sustainabilty point.

## Discussion

The exceptional stability of the checkerboard pattern has to do with the fact that in this state the decoherence among neighboring habitat patches is maximal. To understand this we briefly review some elements of previous studies.

A generic mechanism that leads to sustainability in spatially structured populations has been discovered recently [35],[8] in the context of a two-patch system. The basic ingredients needed for its applicability are migration, stochasticity and an unstable dynamic. (Abta and Shnerb [8] have discussed other stabilization mechanisms that depend on spatial heterogeneity of the local dynamic or, for victim-exploiter systems, on the difference in the migration rates of the species; these attributes do not exist in the models discussed here).

**Figure 8:**
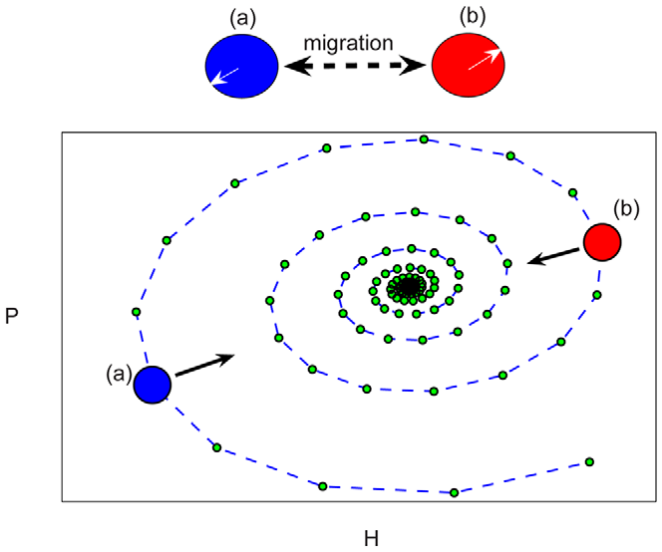
Decoherence as a stabilizer on spatial domains. An illustration of a two-patch system (up), where the intra-patch dynamics on each follows the deterministic Nicholson-Bailey model, as described by Eqs. 11. Here 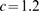, 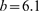 and 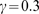. When the system is initiated close to the unstable fixed point, the orbit spirals out (bottom panel, 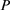 is the parasite density and 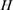 the host density, where the values correspond to NB map are represented by green circles. The dashed line connecting consecutive generations). The amplitude of oscillation grows until one of the species undergoes extinction. If the two patches dynamic is incoherent it is possible to find patch (A) in the state represented by the blue circle, while patch (B) is in the green state. Under these conditions the density of both hosts and parasitoids is larger on (B), thus density independent migration causes a net flow of individuals from (B) to (A). As indicated by the arrows the local communities grow on (A) and shrink on (B) as a result of migration, thus the whole system flows inward towards the coexistence fixed point. This caricature shows that migration is indeed a stabilizer if the time evolution of adjusting spatial patches is incoherent. To avoid diffusion induced synchronization, however, some source of noise is necessary.

In order to grasp the essence of the stabilizing mechanism, let us look at Figure 8. For a simple victim-exploiter 2-patch system, this figure emphasizes that *if* the oscillations on these two patches are incoherent, *then* migration between patches drives the whole system inward toward the coexistence fixed point, yielding sustained oscillations. However, one should bear in mind that dispersal itself tends to reduce population gradients and induces synchronization. In order to gain stability, the migration among patches should be weak enough to allow for noise-induced desynchronization, yet strong enough to stabilize incoherent patches. As discussed in [8], this general statement is valid for any unstable model that supports oscillations close to the unstable fixed point, these oscillations appear naturally in victim-exploiter ecologies [36]. The logistic map (and other unimodular maps like Ricker) also belong to the same class, as the population spirals out of the unstable fixed point [37].

Stable orbits, thus, may appear due to the presence of noise. The role of noise is to perturb the system from its fully synchronized phase. Once this perturbation happens, the differences between patches are amplified by the underlying unstable dynamic. This yields an effective decoherence between patches and, as a result, the dynamic stabilizes.

**Figure 9:**
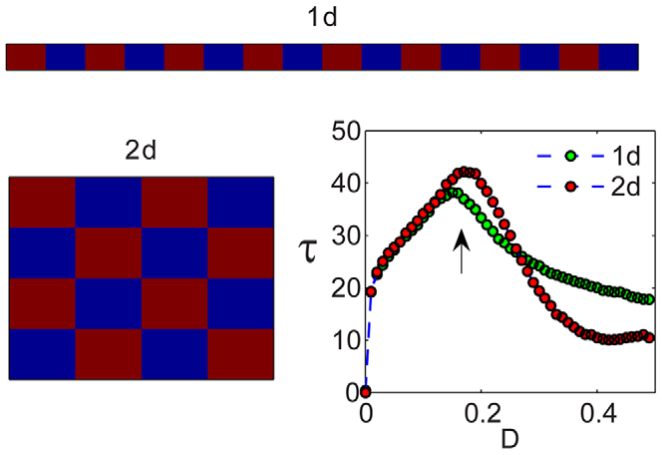
Checkerboards. Average time to extinction (lower right, arb. units) vs. migration rate for one-dimensional array of 16 patches (green) and for 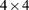 configuration (red). The persistence time peaks are close to each other, and the corresponding spatial configurations are both of a checkerboard type. For an odd number of patches (see Video S2 in the Text S1), the optimum is also in the checkerboard region, with a single defect. In the high migration region, the one-dimensional configuration is preferred, as the weaker coupling avoids synchronization. A representative snapshot of the spatial arrangement is shown for one dimensional (top) and two dimensional (left) arrays, both with the optimal value of 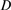 indicated by the vertical arrow.

Based on this insight, we suggest that the optimal persistence is always achieved at the point of maximum decoherence. The basic unit is a two-patch model in the “up-down” phase, and the whole system should be tiled with these dominoes in a checkerboard array that allows for maximum rescue effect. As demonstrated in Figure 9, this conjecture explains the optimal patterns for larger arrays in one and two dimensions. The attractive up-down orbit of the deterministic model and the optimal persistence of the stochastic dynamics coincide, as both manifest the point of maximum efficiency of the stabilizing mechanism [35],[8].

As shown above, the very same result holds for different dynamics that acquire stability through spatial structure, like the Ricker map considered by [12] and the classic Nicholson-Bailey model [38] for host-parasitoid dynamics.

The results of the *Drosophila* experiment [12] also support our conjecture. Although global extinction has not been observed during the experiment, the authors have quantified the constancy stability of the metapopulations by measuring the amplitude of fluctuation in population size over time. This is equivalent to the second method for estimating persistence time explained in the Materials and Methods section below. For the most persistent scenario (optimal migration) the mean nearest neighbor cross-correlation was negative, indicating that the system is indeed in the checkerboard state.

All these considerations fail when the number of individuals per site becomes extremely large (the system follows the deterministic dynamics and the stability of an orbit is governed by the Lyapunov exponent) or small (where synchronization is no longer important and migration always helps). These limits are discussed below.

Within the general framework suggested by Earn, Levin and Rohani [3], our results admit a wide scope of implications. Once the density-dependent local dynamic of the population is known - e.g., by estimating the maximum fecundity parameter or by retrieving the recruitment curve for a well-mixed population (see, e.g., the use of this technique in an annual plant metapopulation experiment [13]) - one may use this deterministic, spatially explicit dynamic to find out which migration rate corresponds to the checkerboard state. This may be used for the design of conservation corridors and for evaluating the impact of habitat fragmentation. For the opposite effect, it may also help in the eradication of infectious diseases. Even without any knowledge of the local dynamics, tracing the patch density vs. time allows one to recover the correlations between neighboring patches; sustainability is optimal when neighbors' correlation reaches its minimum value.

The checkerboard solution manifests itself even if the topology of the system does not allow for “perfect” partition, as in the case of an odd number of patches or an imperfect lattice. As demonstrated in the Video S2, the system develops a local defect that “screens” the problematic region while the rest of the plane is covered by a checkerboard pattern. We have already carried out a preliminary study of other topologies, like equal coupling systems, for which dispersing individuals are equally likely to move to any of the other patches, random networks, triangular lattice etc. Our results suggest that the persistence time is maximal when the system reaches the most incoherent state, which in some aspect resembles the checkerboard strategy; we will return to this issue elsewhere.

## Materials and Methods

### Simulations

The numerical procedure used along this work is a generic individual-based generalization of the deterministic approach for coupled map lattice [39]–[41].

A very similar island model has been used by Hamilton and May [42] who have considered the evolution of dispersal rates for a population with spatial structure and kin competition. However, the model of Hamilton and May, as well as other studies of the persistence of a metapopulation, neglects the demography of the local population: a habitat patch is either occupied or extinct. Under these conditions; the stochastic dynamics is equivalent to a contact process [22]–[24], and the persistence receives only benefit from an increase of the migration rate. To say it another way, simple extinct/occupied dynamics supports, in the deterministic (large 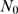) limit, an attractive fixed point with a finite population density. Such a system goes extinct in the presence of stochasticity due to large fluctuations; since larger dispersal acts to decrease the amplitude of these fluctuations it must be advantageous for its persistence.

On the contrary, here we consider the case where in the deterministic limit, the dynamics are unstable (chaotic or otherwise extinction-prone), and thus stochasticity induces fluctuations, and their interference with the spatial structure plays a crucial role in the persistence of the population.

We assume a population dynamics with nonoverlapping generations, where any generation involves two consecutive steps. The first step involves the “local reaction” (birth, death, competition etc., at which any patch is affected only by the local population), and the second is the density independent “migration” (dispersal) step, where individuals are allowed, with a certain probability, to leave their local community and migrate to another patch. No “dispersal cost” is introduced, so any emigrant reaches its chosen destination.

In the reaction step, the number of agents on a patch at the 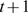 generation, 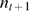, is determined by 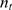. If the numbers of agents are very large (e.g., if one deals with a bacterial system), it is possible to neglect the discrete character of the system, as the effect of demographic stochasticity falls like 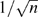. Under these conditions, one can write down a simple map of the form:
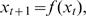
(5)where 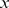 is the population density. Along this work we deal with two particular examples of (5), namely, the logistic map:
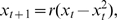
(6)and the Ricker map:
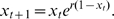
(7)


Both maps are chaotic, and after a while the system reaches population levels that are very close to zero. The deterministic formalism has no problems with that: since 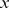 is always above zero, the population survives forever. We know, however, that this is wrong. As the population is made of discrete individuals, 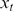 too close to zero corresponds to no individuals at all, in which case the dynamics should halt (this is the “absorbing state” in the stochastic processes terminology). To consider the effect of extinction, one should generalize the deterministic dynamics to include the effect of demographic stochasticity. This is done here by using:
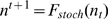
(8)where 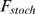 is a stochastic process that converges to its deterministic equivalence (5) in the large population limit. For the logistic map:
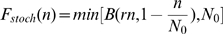
(9)and for the Ricker map:
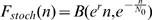
(10)where 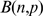 stands here, for the sake of brevity, for a number taken from a binomial distribution (i.e., for 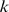 where the chance to get 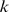 successes from 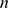 trials is given by the binomial distribution). In the large 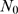 limit, the fluctuations around the mean are negligible, and since the mean of 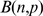 is 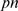, these maps converge to their corresponding deterministic values. In order to facilitate the numerics, we have chosen the value of 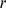 such that the first argument of the binomial distribution will be an integer (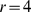 for the logistic map, 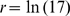 for the Ricker), but it is easy to generalize (9) and (10)) to the case of noninteger values of 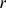.

After the reaction step, a migration step takes place. In the deterministic limit, a fraction 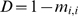 of 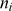 (the population on the i-th site) is subtracted from any site population, and is divided between all possible destinations. In the individual-based model, any individual on the 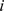-th site is chosen to emigrate with a chance 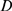, and it then it chooses its destination randomly; for a one dimensional chain it will arrive at the left or at the right neighboring patches with probability 1/2. In order to avoid an artificial drift, the migration takes place via a parallel update scheme, and the site population is updated only after the whole diffusion cycle is completed (failing to do so, one may choose an individual to migrate from the first to the second site, then choose again the same individual to jump from the second to the third patch; this introduces a residual drift in the direction along which the updates take place).

Another example we consider here is the non-chaotic (yet extinction-prone) Nicholson-Bailey [38] model for host-parasitoid interaction. Here there are two species, the host 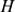 and the parasitoid 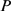, that on a single patch satisfy the deterministic map:
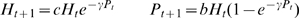
(11)where 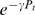 is the escape probability. In the model with demographic stochasticity, 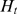 and 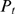 are integers. To simulate the local dynamics the number of uninfected hosts, 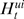, at a certain generation is given by:
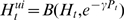
(12)and the rest of the hosts are infected. Any uninfected host produces 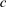 offspring in the next generation, while an infected host yields 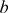 parasitoids. The migration procedure is the one presented above where it is now applied separately to the host and to the parasite.

### Estimating persistence time

Along this work we have used three procedures in order to estimate 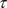, the persistence time of a system. As discussed in [35],[8], the distribution of lifetimes is exponential with an average 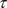 (see Text S1). This has to do with the appearance of an attractive manifold, as opposed to the broad distribution of lifetimes at criticality as discussed in[43].

#### 1. Direct measurement of 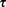


If the system reaches extinction on reasonable timescales, we can simply average over the time to extinction obtained from repeated runs of the simulation with random initial conditions and different histories. The result is 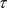, the average time to extinction.

#### 2. Stability of the attractive manifold

To implement this method, one assumes that the population fluctuates, more or less normally, around 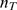, the total population that corresponds to the attractive orbit (see Figure 3 above). The variance of these fluctuations is 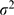, and the average time to extinction should be proportional to the chance for the total population to be zero. Assuming Gaussian fluctuations, this implies that the extinction time scales exponentially with 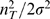 [44],[45],[46]. This method allows one to estimate the stability of a large system, when the time to extinction is large.

#### 3. Averaging over the deterministic dynamics

Here we did not use the stochastic simulations at all. Instead, it is assumed that the demographic fluctuations effectively kicked the system away from the stable orbit, into a random location (picked with uniform distribution among all possible states) in the phase space. This chosen point becomes a new “initial condition” that flows, in the deterministic limit, back to the attractive orbit along a transient trajectory. The chance of extinction during this transient is proportional to the minimum over time of the total population, 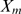 (see caption of Figure 3) for these initial conditions. The time to extinction is thus proportional to the average of 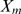 over the whole phase space.

**Figure 10:**
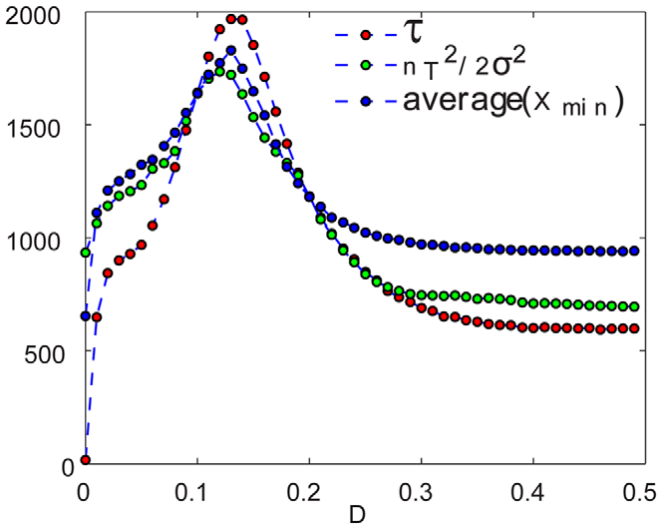
Three methods for finding extinction times: a comparison. The value of 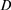 which optimizes the time to extinction shows a complete agreement between the methods. We have used the exponent of 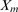 and 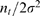 in order to put all curves on the same scale, as explained in the text. Here 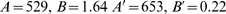.

In Figure 10 these three technique are compared for the two-patch logistic system with 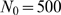. Roughly speaking, one should expect the time to extinction to scale exponentially with the size of the minimal population 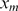, and/or with the overlap of the normal distribution characterized by 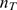 and 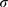 with the zero population state [47] (these, of course, are only rough estimates, see [44],[45],[46] for a more accurate treatment). Within this framework, the estimated extinction times for the second method were calculated from 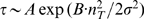, and for the third method 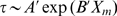, where the parameters 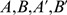 were extracted by fitting the data at two points from both sides of the peak. One can see that the maximum persistence appears at the same migration rate and that the deviations among graphs are of order 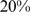, which is way beyond what is expected from such a crude estimate.

### The Extreme Limits: Dense and Dilute Populations

There are two extreme cases of a too large and too small noise, where the checkerboard strategy fails. In the weak noise limit (that corresponds to the large 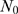 case of the agent-based system) the dynamics is very close to the deterministic one and the results of the deterministic modeling most hold. It turns out that the size of 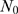 needed to reach this limit is huge, and any ecosystem (except, maybe, bacterial colonies) is far from this extreme. In particular, local extinction happens only if the deterministic dynamics takes the population to very small values, between zero and 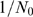. This is a relevant process only in the weak migration regime (the rate of recolonization approaches zero) or in the fully synchronized case; otherwise, extinction simply never happens in this regime.

**Figure 11:**
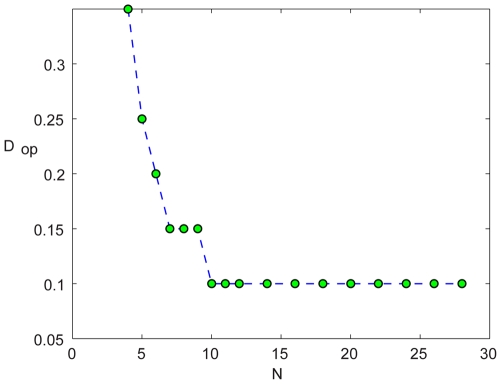
The dilute limit: stronger migration - higher persistence. The optimal migration rate (the one that yields the maximal time to extinction) 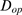, vs. 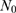 for the two-patch Ricker map. For 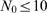 the optimal migration is not in the checkerboard region anymore.

The other limit, that of large noise, appears when 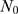 is very small. In this case the rate of local extinctions is so high that the real degrees of freedom of a habitat patch are simply occupied or empty, and coherence among patches makes no difference. Our island model with complex dynamics becomes equivalent, in the strong noise limit, to a contact process. As explained above, in that case the higher the migration rate, the more sustainable is the metapopulation, and thus the optimal migration rate grows as 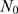 approaches one. This phenomenon is demonstrated in Figure 11.

## Supporting Information

Text S1 First, in the main only demographic stochasticity was considered. Here we show that the same results hold for a system subject to both environmental and demographic stochasticity. Second, in the main part of the paper only the average time to extinction was presented. Here we show the whole probability function and confirm that it is exponential distribution.
(0.09 MB PDF)

Video S1 As the migration parameter D increases, the fractal map showing Xm for any possible initial condition of a two patch system is changes (lower right). In the lower left panel the orbit diagram is updated for any given D. the upper panel shows the average of Xm over all possible initial states; it yields the bell-shape with the peak at the optimal sustainability point, as explained in the text.
(8.58 MB MPG)

Video S2 The individual based Ricker dynamic is simulated on a 10×10 lattice (periodic boundary conditions), with a “defect” (inaccessible sits, dark blue) in the middle. The movie present consecutive snapshots of the density of particles, color coded as indicated by the color bar, at the optimal migration point. One realizes that the system reaches the checkerboard state, with a single moving imperfection localized close to the defect.
(5.82 MB MPG)
