# Supplementary material for: Optimizing Metapopulation Sustainability through a Checkerboard Strategy
Source: PLoS Comput Biol. 2010 Jan 22;6(1):e1000643. doi: 10.1371/journal.pcbi.1000643 (PMC2798954; doi:10.1371/journal.pcbi.1000643)
Supplement: Text S1 — First, in the main only demographic stochasticity was considered. Here we show that the same results hold for a system subject to both environmental and demographic stochasticity. Second, in the main part of the paper only the average time to extinction was presented. Here we show the whole probability function and confirm that it is exponential distribution. (0.09 MB PDF) [file pcbi.1000643.s001.pdf]

Supplementary Material: Optimizing metapopulation sustainability through a checkerboard strategy

Yosi Ben Zion[1,*], Gur Yaari[2], Nadav M. Shnerb[1]

1Department of Physics, Bar-Ilan University, Ramat-Gan 52900,Israel

2 Department of Ecology and Evolutionary Biology, Yale University, New Haven, Connecticut 06520, USA

∗ E-mail: benzioy@mail.biu.ac.il

The effect of environmental stochasticity

Along the paper we consider only one type of noise in the system, namely, the demographic stochasticity. The advantages of this type of noise is that it allows for a natural definition of the extinction as the inactive (”absorbing”) state of zero population, and that it exists even in controlled experiments. It is well known, however, that other types of noise do affect any realistic ecosystem in nature. In order to examine the impact of other types of noise, and the robustness of the checkerboard strategy, we consider here a single example; extensions of this study will be presented in subsequent publication.

Starting with a simple two-patch Ricker system, for which the deterministic dynamics is described by the map:

$$\begin{aligned} x_1^{t+1} &= (1-D)f(x_1^t) + Df(x_2^t) \\ x_2^{t+1} &= (1-D)f(x_2^t) + Df(x_1^t) \end{aligned} \tag{1}$$

where D is the proportion of individuals from a patch to disperse and f is the reproduction function. The Ricker map is $f(x_i^t) = x_i^t e^{(r(1-x_i^t/n_0))}$ where r is the maximum fecundity and n_0 is the carrying capacity.

The affect of demographic stochasticity on this system is described in the main text. Here we subjected the system to both demographic and environmental noise and showed that the checkerboard strategy still holds.

Following [1, 2] we consider the case where the maps are modulated by stochasticity ξ

$$\begin{aligned} x_1^{t+1} &= \xi_1[(1-D)x_1^t e^{(r(1-x_1^t/n_0))} + Dx_2^t e^{(r(1-x_2^t/n_0))}] \\ x_2^{t+1} &= \xi_2[(1-D)x_2^t e^{(r(1-x_2^t/n_0))} + Dx_1^t e^{(r(1-x_1^t/n_0))}]. \end{aligned} \tag{2}$$

The noise terms ξ are from uniformly distributed random numbers in the range $1w, 1+w$, where $w = 0.2$.

Eqs. 2 describe a system with no demographic stochasticity, and the size of the population may take noninteger values. At the end of any time step Eqs. 2 yield two numbers, x_1 and x_2, which are the expected average population on the corresponding island. To make this model individual-based the following procedure has been adopted: at each time step two integers, n_1 and n_2, were drown at random from a Poissonian distribution with average x_1 and x_2; these integers are then feeded back as the population size for the next iteration of 2.

We simulate the system and measured the persistence time as a function of the the dispersion rate for two different noise terms: perfectly correlated stochasticity and stochasticity with no correlation at all [1,3]. The results are presented in Fig 1. In both cases the sustainability peaked at the same dispersion parameter, at the same point it appears for the case of pure demographic noise (see, fig. 4 of the main text that present the result for identical system without environmental stochasticity), as before, the peak appears where the deterministic map [Eq. 1] supports period orbits (the up-down solution).

One can easily see that a reduction in the absolute value of the life-time in the correlated case is a result of the effect of synchronization in correlated noise [4, 5].

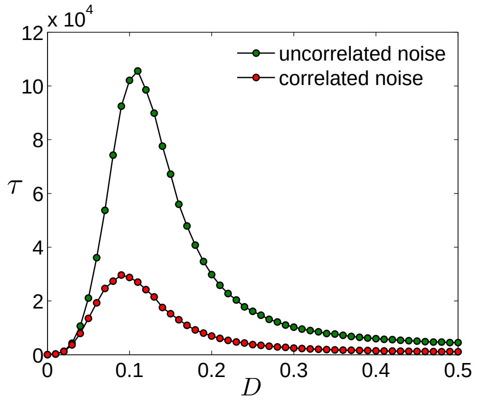

Figure 1. A two-patch system of coupled Ricker maps with environmental stochasticity. The average time-to-extinction of the individual-based dynamics [Eq. 2] shows persistence peaks in the region of up-down for $N_0 = 20$ and $r = 2.833$, to be compared with Fig. 4 of the main text.

Distribution of the extinction time

As expected for the case of extinction in the presence of an attractive manifold in the feasible regime [6–8] the distribution of extinction times is exponential, as shown in fig. 2.

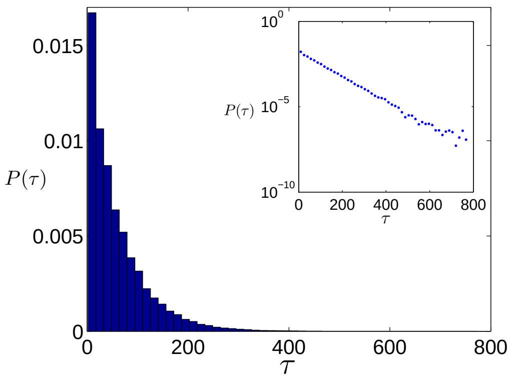

Figure 2. Typical distribution of the time-to-extinction : The system shows exponentially distribution. The insight shows log-normal plot. The distribution simulated for a two-patch system of coupled Ricker maps.

References

1. Ranta E, Lundberg P, Kaitala V (2006) Ecology of Populations. Cambridge UK: Cambridge Univ. Press.

2. Ranta E, Kaitala V (2006) Comment on "stability via asynchrony in drosophila metapopulations with low migration rates". Science 314: 420.

3. Ripa J, Lundberg P (1996) Noise colour and the risk of population extinctions. Proceedings of the Royal Society B 1377: 1751-1753.

4. Moran PAP (1953) Noise colour and the risk of population extinctions. Aust J Zool 1: 291298.

5. Sutcliffe OL, Thomas CD, Yates TJ, Greatorex-Davies JN (1997) Correlated extinctions, colonizations and population fluctuations in a highly connected ringlet butterfly metapopulation. Oecologia 109: 235.

6. Elgart V, Kamenev A (2004) Rare event statistics in reaction-diffusion systems. Phys Rev E 70: 041106.

7. Assaf M, Meerson B (2006) Spectral theory of metastability and extinction in birth-death systems. Phys Rev Lett 97: 200602.

8. Kessler DA, Shnerb NM (2007) Extinction rates for fluctuation-induced metastabilities: A real-space wkb approach. J Stat Phys 127: 861.
